# Characterising particulate matter source contributions in the pollution control zone of mining and related industries using bivariate statistical techniques

**DOI:** 10.1038/s41598-020-78445-5

**Published:** 2020-12-07

**Authors:** Sirapong Sooktawee, Thongchai Kanabkaew, Suteera Boonyapitak, Aduldech Patpai, Nirun Piemyai

**Affiliations:** 1 Environmental Research and Training Center, Department of Environmental Quality Promotion, Ministry of Natural Resources and Environment, Pathumthani, Thailand; 2 Department of Sanitary Engineering, Faculty of Public Health, Mahidol University, Bangkok, Thailand; 3 Center of Excellence on Environmental Health and Toxicology, Bangkok, Thailand

**Keywords:** Environmental impact, Atmospheric dynamics

## Abstract

Na Phra Lan Subdistrict is a pollution control zone with the highest PM_10_ level in Thailand. Major mobile and industrial sources in the area are related to stone crushing, quarrying and mining. This study used statistical techniques to investigate the potential sources influencing high PM_10_ levels in Na Phra Lan. Hourly PM_10_ data and related parameters (PM_2.5_, PM_coarse_ and NO_x_) from 2014–2017 were analysed using time series, bivariate polar plot and conditional bivariate probability function (CBPF). Results of diurnal variation revealed two peaks of PM_10_ levels from 06:00–10:00 and 19:00–23:00 every month. For seasonal variation, high PM_10_ concentrations were found from October to February associated with the cool and dry weather during these months. The bivariate polar plot and CBPF confirmed two potential sources, i.e., resuspended dust from mobile sources close to the air quality monitoring station (receptor) and industrial sources of mining, quarrying and stone crushing far from the station on the northeast side. While the industrial source areas played a role in background PM_10_ concentrations, the influence of mobile sources increased the concentrations resulting in two PM_10_ peaks daily. From the study results, we proposed that countermeasure activities should focus on potential source areas, resuspended road dust from vehicles and the industrial sources related to quarrying and mining, rather than distributing equal attention to all sources.

## Introduction

Particulate matter (PM) in the air is an important issue for sustainable development. The United Nations anticipates achieving the Sustainable Development Goals (SDGs) by 2030. Annual mean levels of PM_2.5_ and PM_10_ are indicators of goal 11 of the SDGs that aims to make cities and human settlements inclusive, safe, resilient and sustainable^1^. The recommended PM_10_ values from the guidelines of the World Health Organisation (WHO) are 50 and 20 µg/m^3^ for 24-h and annual averages, respectively^2^. Exposure to high level of PM causes several adverse health problems both acute and chronic effects, e.g., bronchitis, asthma, respiratory and cardiovascular diseases^3–5^, and diabetes^6^ that subsequently induce the years of human life lost^7^. Regarding populations in developing countries, around 9 of 10 individuals were found to have a high possibility of being exposed to higher levels of PM than those specified in WHO guidelines^8^. Recently, Taneepanichskul et al.^9^ found an increased risk of mortality in 12 provinces of Thailand attributable to increased PM_10_ concentrations, particularly during the winter months (November to February).

For Thailand, the PM_10_ levels in Na Phra Lan Subdistrict, Saraburi Province, have reached the highest in the country for a few decades^10^. Since 1997, air quality monitoring station has measured PM_10_ concentration in the area. The maximum 24-h average in 1997 was 693 µg/m^3^ and 37% of observed times (54 of 147) exceeded the National Ambient Air Quality Standard (NAAQS), i.e., 120 µg/m^3^ for 24-h average as posted in the http://air4thai.pcd.go.th website of the Pollution Control Department (PCD) of Thailand. Therefore, the Thai government has designated Na Phra Lan Subdistrict as a “Pollution Control Zone” since 2004 and the local governor was given the authority to implement specific action plans to mitigate the problem^11^. From 2012 to present, the tendency of PM_10_ concentrations in other parts of the country revealed a decreasing trend, except for the area of Na Phra Lan Subdistrict^10^.

The PM_10_ sources in Na Phra Lan Subdistrict were observed to be mainly from industrial processes related to quarrying activities such as mineral processing plants, crushed stone plants, and stone mines^12–14^. Nonetheless, not only were the industrial sources, but also the resuspended road dust from transporting mining products identified as major sources of PM_10_^14^. Related studies using the source dispersion model have indicated that PM_10_ levels at receptors were generated mainly from line source emissions of resuspended road dust, followed by area sources related to mining industries^13,14^. However, dispersion model utilised estimated emission sources and simulated meteorological parameters. Various assumptions of emission estimation have been applied to specify activity data, emission factors, and temporal and spatial allocations of emission inputs that could create uncertainties in the prediction. Simulated meteorological parameters were also downscaled and processed using a large-scale meteorological input from reanalysed datasets with 1 × 1° resolution from the National Center for Environmental Prediction. High variable meteorological parameters such as wind speed and wind direction from model simulation were seen to be not in good agreement with the observation. In addition, a selected episode of short period for model simulation as 2 days of HYPACT in winter and rainy seasons^13^, and normally a 1-year basis of AERMOD run without model result evaluation with the reference monitoring data^14^ could pose a level of uncertainties. In addition, no relationship among temporal variations of PM_10_, meteorological parameters, and emission source contribution and locations has been clearly described in Na Phra Lan area. Other studies have also reported that the dispersion model result did not provide adequate information of locations and directions of source contributions to the receptors regarding specific temporal variation and, to some extent, the incomplete information of the sources and their emissions was regarded as the model limitation^15,16^. Therefore, this study aimed to provide further information to characterise PM_10_ source contributions, their diurnal emissions and their locations, using integrated statistical techniques: bivariate polar plot and conditional bivariate probability function (CBPF) for long-term actual monitoring data from 2014–2017. Results are anticipated to be useful to policymakers to appropriately reduce PM_10_ from the accurate and potential sources in the Na Phra Lan pollution control zone.

Statistical techniques using bivariate polar plot and CBPF provide benefits to identify source characterisation in a complex emission area and display filtering air pollution data associated with wind speed and direction, and time of day; hence, they provide directionality of sources in a specific time period^15,17–19^. CBPF has proven to be useful to identify the direction of major emission sources in rural New York State, US from December 2004 to December 2008^20^. Uria-Tellaetxe and Carslaw^15^ conducted their study in the North Lincolnshire Unitary Area, UK reporting that CBPF was an effective tool to detect source direction of NO_x_ and SO_2_ while bivariate polar plot provided information on source dispersion. Combining these statistical techniques with the area map, the specific sources of emissions could then be identified^15^. In addition, the bivariate polar plot and CBPF were used as effective tools to assess and manage air quality in various regions including Belgrade, Serbia^21^, Lahore, Pakistan^22^ and 16 cities in south central Chile^23^.

## Study area and methods

**Figure 1:**
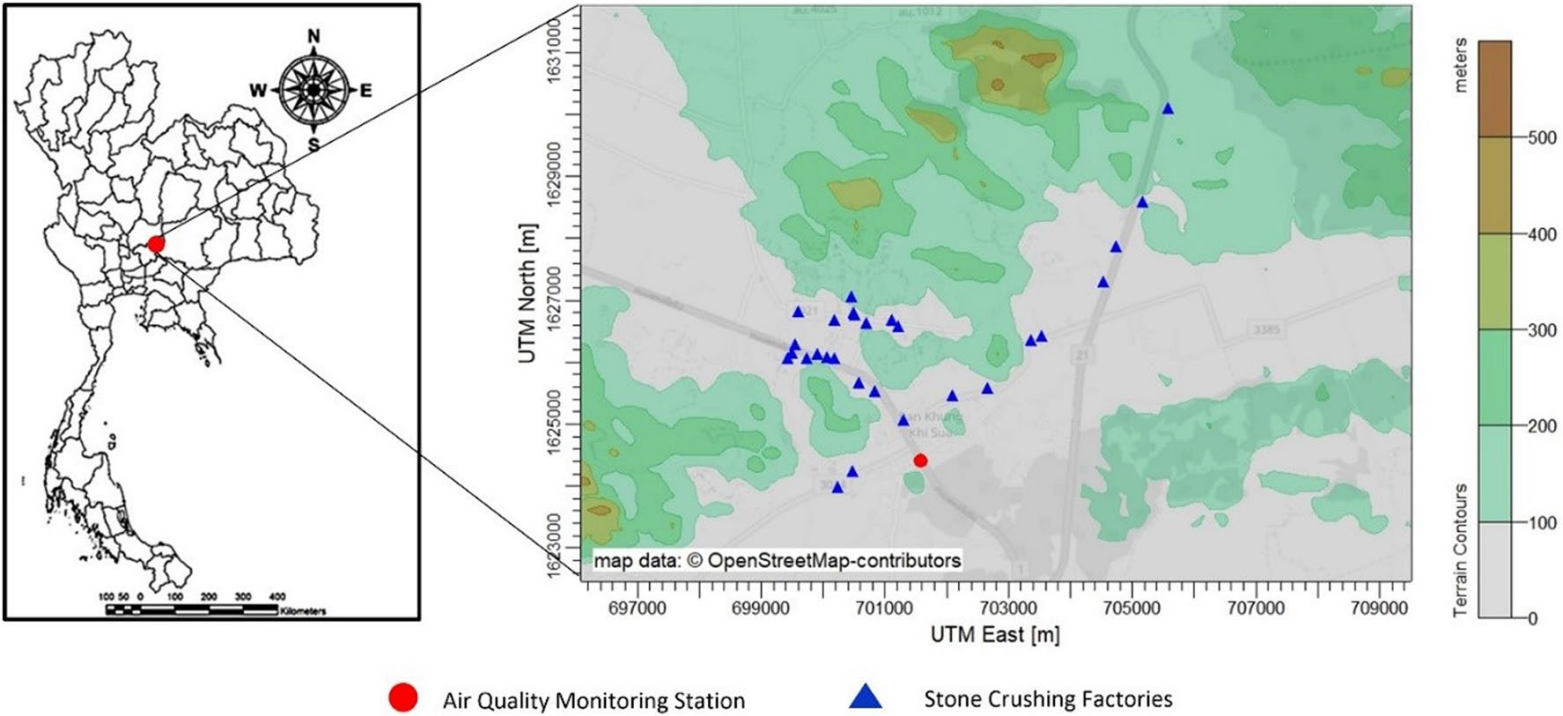
Study area and the location of air quality monitoring station located in Na Phra Lan Subdistrict (a map was created using AERMOD View 9.8.1, www.weblakes.com/products/aermod/).

Na Phra Lan Subdistrict is a part of Chaloem Phra Kiat District, Saraburi Province located in central Thailand. This subdistrict accommodates various activities related to PM_10_ emission, e.g., mining, quarrying, and stone crushing. The Department of Primary Industries and Mines reported on the website, http://www1.dpim.go.th, that 33 stone crushing plants were located in Na Phra Lan Subdistrict of 53 plants in Saraburi Province, and 7 limestone concessions in Na Phra Lan Subdistrict of 20 concessions in the province. The PCD reported that the maximum 24 h average concentration of PM_10_ was 268 µg/m^3^ from 2014–2017 whereas the 24-h average NAAQS of PM_10_ was 120 µg/m^3^. In terms of interannual variation, the annual average PM_10_ was around 100 µg/m^3^, and higher than the annual NAAQS of PM_10_ (50 µg/m^3^). The air quality monitoring station operated by PCD is located in the area of Na Phra Lan police station close to the main road, Pahonyothin Road. A map in Fig. 1 presents the location of the air quality station and Na Phra Lan Subdistrict. Hourly PM_10_, PM_2.5_, NO_x_, wind speed and direction, rain and relative humidity (RH) data from 2014–2017 were observed at this station and used for analysis. Thermo Scientific Model 5014i Beta Continuous Particulate Monitor unit has been used to measure PM_10_ and PM_2.5_, and the 42i model has been utilised for NO_x_ monitoring. For wind speed and direction, rain and RH monitoring, LSI LASTEM instruments have been used. The observed hourly missing data were 2.8%, 32.7%, and 6.9% for PM_10_, PM_2.5_, and NO_x_, respectively. The PM_2.5_ data has been checked that its magnitude should not be greater than PM_10_. The hourly PM_coarse_ was calculated by subtracting the corresponding hourly PM_2.5_ from PM_10_ concentrations.

Data were analysed to visualise the historic variations in PM_10_ concentrations and potential source contributions to understand the air quality situation within the study area. R program^24^ with OpenAir package^25^ was used and the analysis was divided into two main parts. The first part comprised temporal analysis to understand the situation and variation of PM_10_ levels in the study area by examining wind rose, pollution rose, intra-annual variation, and diurnal variation. The second part was analysed using the bivariate polar plot and CBPF to examine the spatial analysis, and identify potential emission sources of PM_10_ contributing to air quality in Na Phra Lan; hence, the monitoring station was used as a receptor point for the study.

Bivariate polar plot is a technique for producing statistical values such as mean value in the polar pattern with radial (r) and angles (θ). For air quality analysis, radial axis (r) and angular axis (θ) were used to represent wind speed and wind direction, respectively. Wind speed and wind direction were divided into small cells. Then, PM_10_ concentration data were distributed into a related cell of wind speed and wind direction. The statistical metrics such as mean value of PM_10_ were calculated and shaded in that corresponded cell of the polar coordinate. The shading surface was smooth with general auxiliary models (GAM) indicated in Eq. (1) as follows:

1 $$\sqrt {C_{i} } = \beta_{0} + s(u_{i} ,v_{i} ) + \varepsilon_{i}$$

where, $C_{i}$ is the pollutant concentration, $\beta_{0}$ is overall mean of response, $s(u_{i} ,v_{i} )$ is the smooth function, *u* and *v* are the wind components: *u* = *ū*.sin(2π/θ) and *v* = *ū*.cos(2π/θ), *ū* is the mean wind speed, $\varepsilon_{i}$ is residual^15^. This technique can be used to determine the potential source of air pollutants such as identify the potential source of various pollutants at the Scunthorpe Town, U.K.^15^, and at Krakow, Poland^26^.

For CBPF, this technique is based on the probability of the observed pollutant concentrations that exceed the threshold set for each range of wind speed and wind direction. CBPF value in each range of threshold concentrations can identify the potential emission sources of pollution that affect the monitoring point^15^. However, CBPF is a receptor model, and its results cannot be used to directly compare with the measurement value which is different from the results given by the source dispersion model^27^. The result obtained from CBPF analysis can be used to compare with a spatial map to determine the consistency of the significant source influencing the level of pollutant at air quality monitoring station^15,22^. CBPF can be calculated using Eq. (2) as follows:

2 $${\text{CBPF}}_{\Delta \theta ,\Delta v} = \frac{{\left. {m_{\Delta \theta ,\Delta v} } \right|_{y \ge C \ge x} }}{{n_{\Delta \theta ,\Delta v} }}$$

where, $m_{\Delta \theta ,\Delta v}$ is a number of sampling data with concentration between the given concentration interval x and y, and within the range of wind speed ($\Delta v$) and wind direction ($\Delta \theta$). $n_{\Delta \theta ,\Delta v}$ is a number of sampling data with any concentrations within the range of wind speed ($\Delta v$) and wind direction ($\Delta \theta$)^15^.

## Results and discussion

### Situation of PM_10_ in Na Phra Lan subdistrict

**Figure 2:**
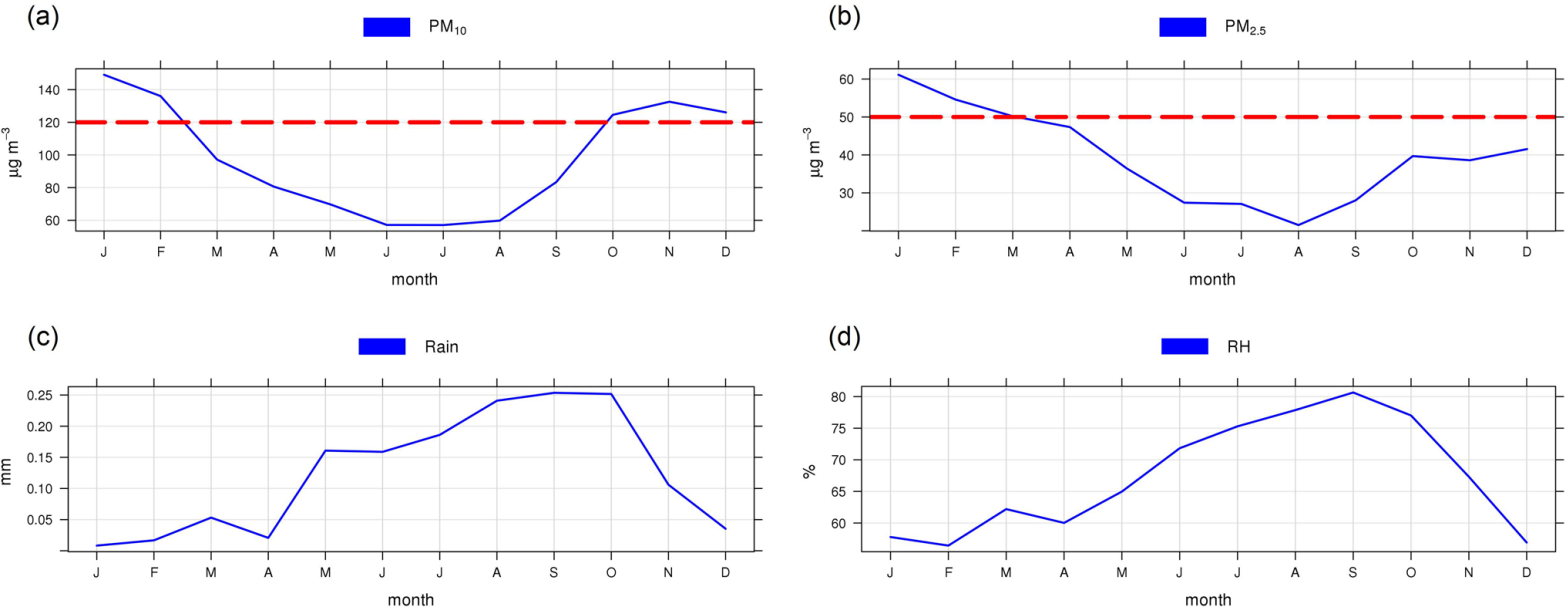
Intra-annual variations of (**a**) PM_10_, (**b**) PM_2.5_, (**c**) rain, and (**d**) RH (relative humidity) (the dash line is the 24-h NAAQS of PM_10_ and PM_2.5_).

To understand the mechanisms that enhance high PM_10_ concentrations in Na Phra Lan Subdistrict, intra-annual variations of PM_10_ were visualised using monthly average analysis. The intra-annual variation revealed that PM_10_ and PM_2.5_ concentrations were higher than the Thailand NAAQS from approximately October to February, and January to February, respectively (Fig. 2). Both PM_2.5_ and PM_10_ were also over the interim target-2 level of WHO air quality guideline for PM: 24-h concentrations, which are 100 and 50 µg/m^3^ for PM_10_ and PM_2.5_, respectively^2^. Variation of the PM changes in relation to seasons; PM becomes high when amount of rain is less, and vice versa for high intensity of rain. The season changes from rainy to winter in late October. The latter season from November to February becomes the dominant mode of weather governing climatic conditions over Thailand and among its neighbours^28,29^. During winter season, the Siberian High is a dominant forcing that results in changes of pressure gradient, temperature, precipitation, wind circulation, and others over Thailand^28,30^. In addition, planetary boundary layer (PBL) is another factor in wintertime affecting air quality. PBL over terrestrial area located in tropical zone of the northern hemisphere becomes lower than other seasons^31^. The height of PBL that performs complete mix of substances emitted into the layer has been known as the mixing height, and is determined by pinpointing the temperature inversion on the vertical temperature profile^32^. Cold air traveling during winter season causes the PBL depth to become shallow resulting in reduction of the mixing height^31,32^, which relates to reduction of air volume utilised for mixing substances and results in an increase of air pollutant concentration if the pollutant mass is constant.

There is a large amount of precipitation and high RH during the wet period (May to October) caused by the summer monsoon^30,33^. Air pollutants are removed by atmospheric processes such as wash-out and less photochemical reactions during rainfall enhancing reduction of PM_10_, PM_2.5_, and gaseous pollutants^34^. We can conclude that intra-annual variations of PM_10_ and PM_2.5_ relate mainly to climatic variation. The PM_10_ and PM_2.5_ concentrations at Na Phra Lan Subdistrict increase during winter season under the strong atmospheric inversion associated with cool air mass and high pressure. Furthermore, seasonal variation does not only result in a change of climatic scalar parameters but also affects a change of wind that is a vector parameter.

**Figure 3:**
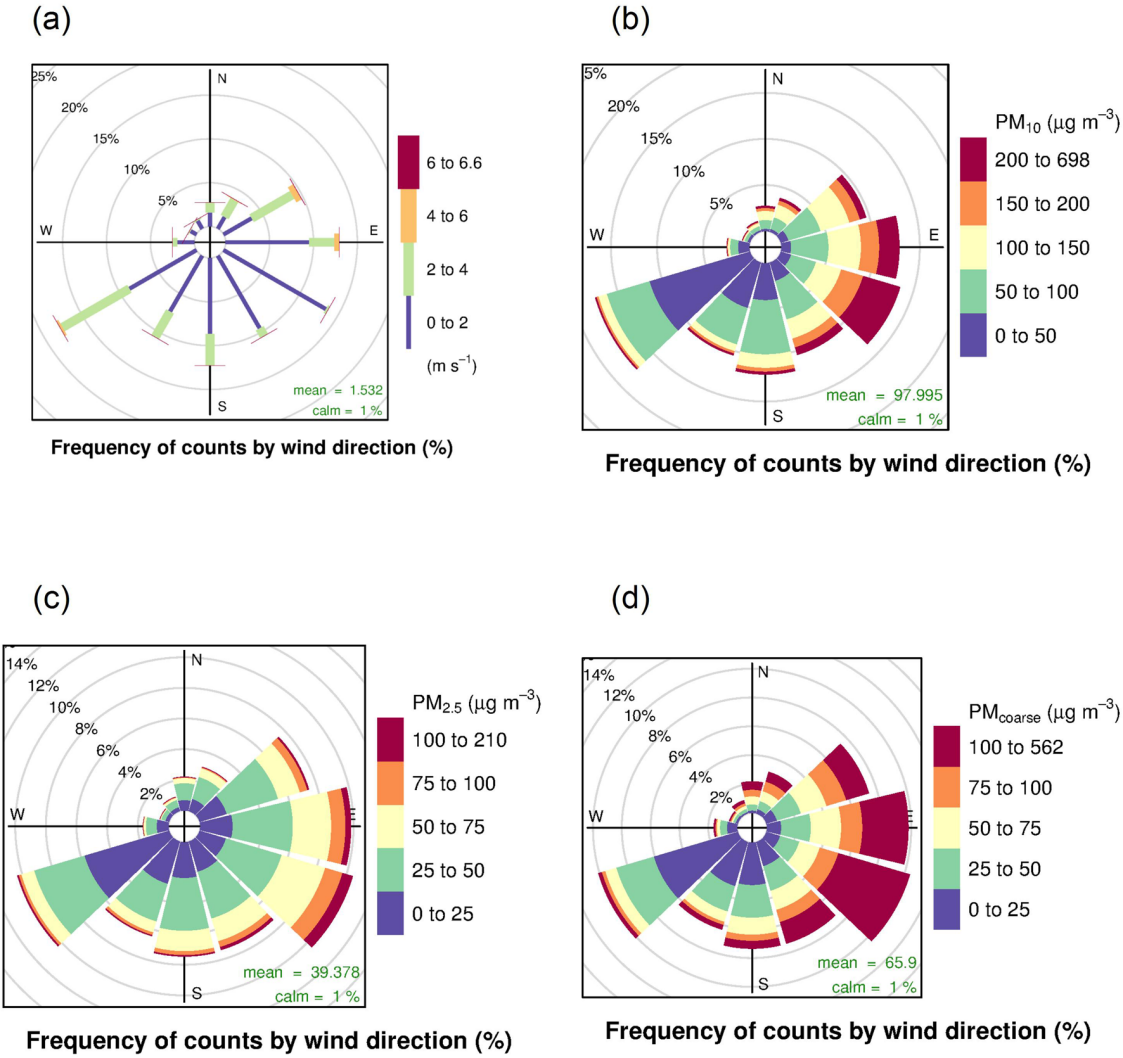
(**a**) Wind rose and pollution roses of (**b**) PM_10_, (**c**) PM_2.5_, and (**d**) PM_coarse_.

During winter, wind circulation over Thailand is governed by the northeasterly wind^28^. This particular meteorological condition is one of the factors resulting in the change of pollution concentrations and plays an important role in leveling up the pollution in the country^35–37^. Wind rose and pollution rose were constructed using data from the air quality monitoring station to investigate associations between the wind and PM_10_ at the receptor. Results revealed that approximately 70% of wind data were mainly from the direction between northeast and southwest (Fig. 3a). The low frequency of northwesterly wind is due to the elevated terrain located at the northern and western areas of the station (see Fig. 1), and it is not the prevailing wind direction of summer and winter monsoons. When considering the pollution rose (Fig. 3b,c), PM_10_ and PM_2.5_ levels, higher than 100 and 50 µg/m^3^, were mainly from the east side of the air quality station (from northeast to southeast). Figure 3d shows the pollution rose of PM_coarse_, which is a main ingredient of PM_10_. High PM_coarse_ concentrations were mainly from northeast to southeast direction, and similar to the PM_10_. Therefore, it is interesting to investigate the PM_2.5_/PM_10_ ratio further because the ratio could help suggest types of PM sources^38^.

**Figure 4:**
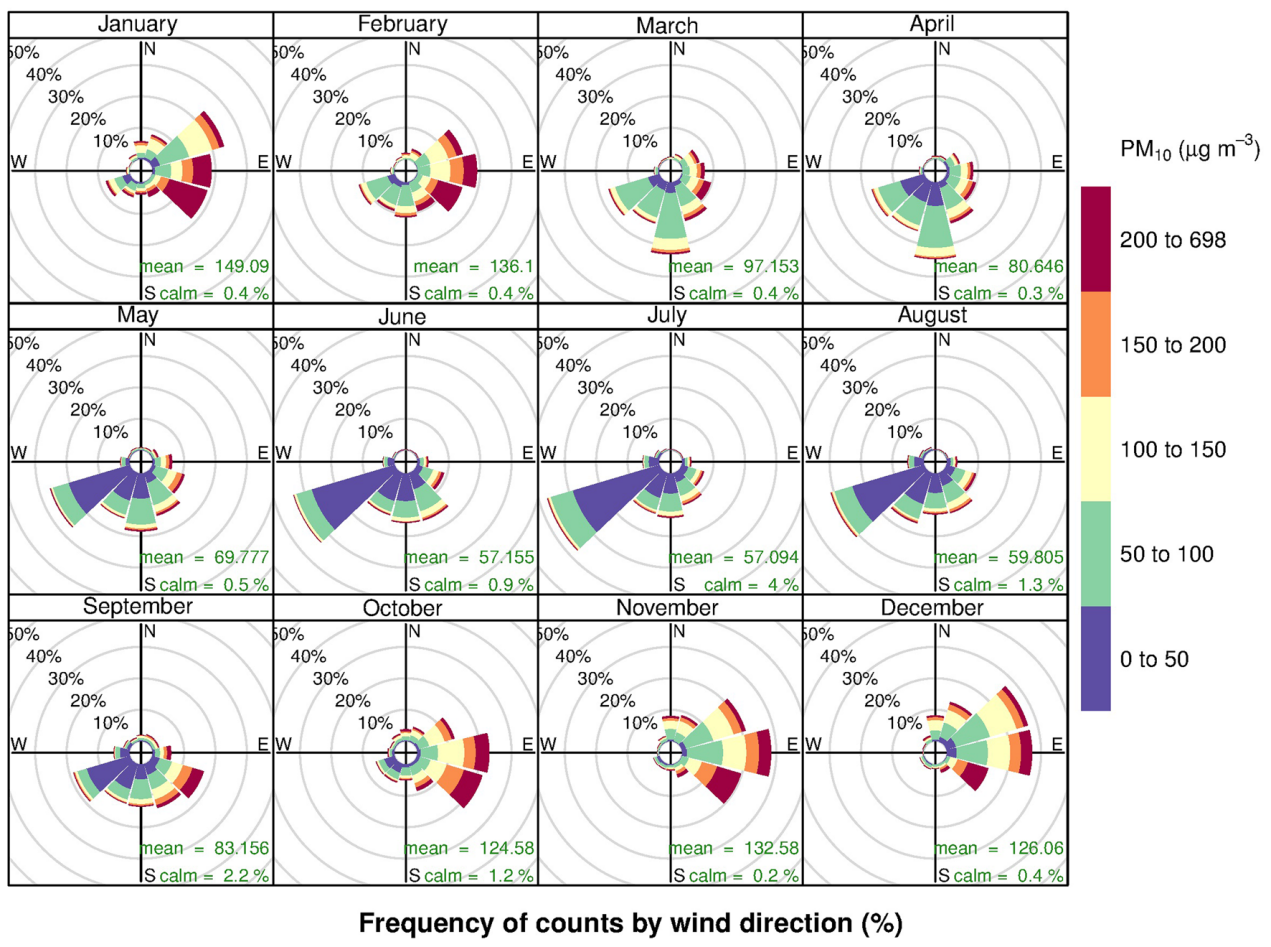
Monthly pollution roses of PM_10_ in Na Phra Lan Subdistrict.

Separated monthly pollution rose plots were then investigated in Fig. 4 to scrutinise the effects of meteorological patterns on the pollution formation. The proportion of high PM_10_ concentrations was presented from October to February with the predominant winds from the direction between northeast and southeast consistent with the overriding winter monsoon. However, with the wind blowing from other directions in other months, the proportion of high PM_10_ concentrations was significantly lower than in winter. The pollution roses suggest that significant PM_10_ sources would be located at the east area during the winter season. Pollution roses of PM_coarse_ were similar to those of PM_10_ in terms of frequency with slightly lower concentrations whereas for PM_2.5_ roses, concentrations were significantly lower than PM_10_ (Figures S1 and S2 in supplementary).

Mean concentrations of PM_10_, PM_2.5_, PM_coarse_, and NO_x_ in Na Phra Lan Subdistrict were 97.98 μg/m^3^, 39.37 μg/m^3^, 65.84 μg/m^3^, and 53.71 ppb, respectively. The mean PM_2.5_/PM_10_ ratio was 0.4 and the correlation of PM_10_ and PM_coarse_ was 0.95, whereas that of PM_10_ and PM_2.5_ was 0.67 at 99 percent confidence level. This revealed that the PM_10_ level in Na Phra Lan Subdistrict would be contributed by PM_coarse_ more than PM_2.5_. The correlations of NO_x_ and PM_10_, and PM_2.5_ were also investigated. The correlation of NO_x_ and PM_10_ was 0.76, and the latter was 0.48. High correlation of NO_x_ and PM_10_ suggests that PM_10_ level would be strongly associated with traffic emissions and its related activities, while the lower correlation of NO_x_ and PM_2.5_ implies that most of the emitted PM_10_ was not from the tailpipe. A similar study in China found that the ratios of PM_2.5_/PM_10_ were 0.617, 0.630, and 0.680 for urban, urban fringe, and suburban^39^. Munir et al.^40^ presented that the PM_2.5_/PM_10_ ratio was greater than 0.6 for urban traffic area. Zhao et al.^38^ suggested that a contribution of more coarse PM sources involved by mechanical processes, e.g. resuspended dust is related to a low ratio of PM_2.5_/PM_10_ (less than 0.5), whereas a high ratio (greater than 0.6) is related to emissions from industrial and vehicular fuel combustion. In this study, although the air quality monitoring station is located near the road, the ratio of PM_2.5_/PM_10_ (0.4) does not represent vehicular fuel combustion in the urban area. The high PM_10_ in the area would be affected by mechanical activities more than emission from the traffic tailpipe.

**Figure 5:**
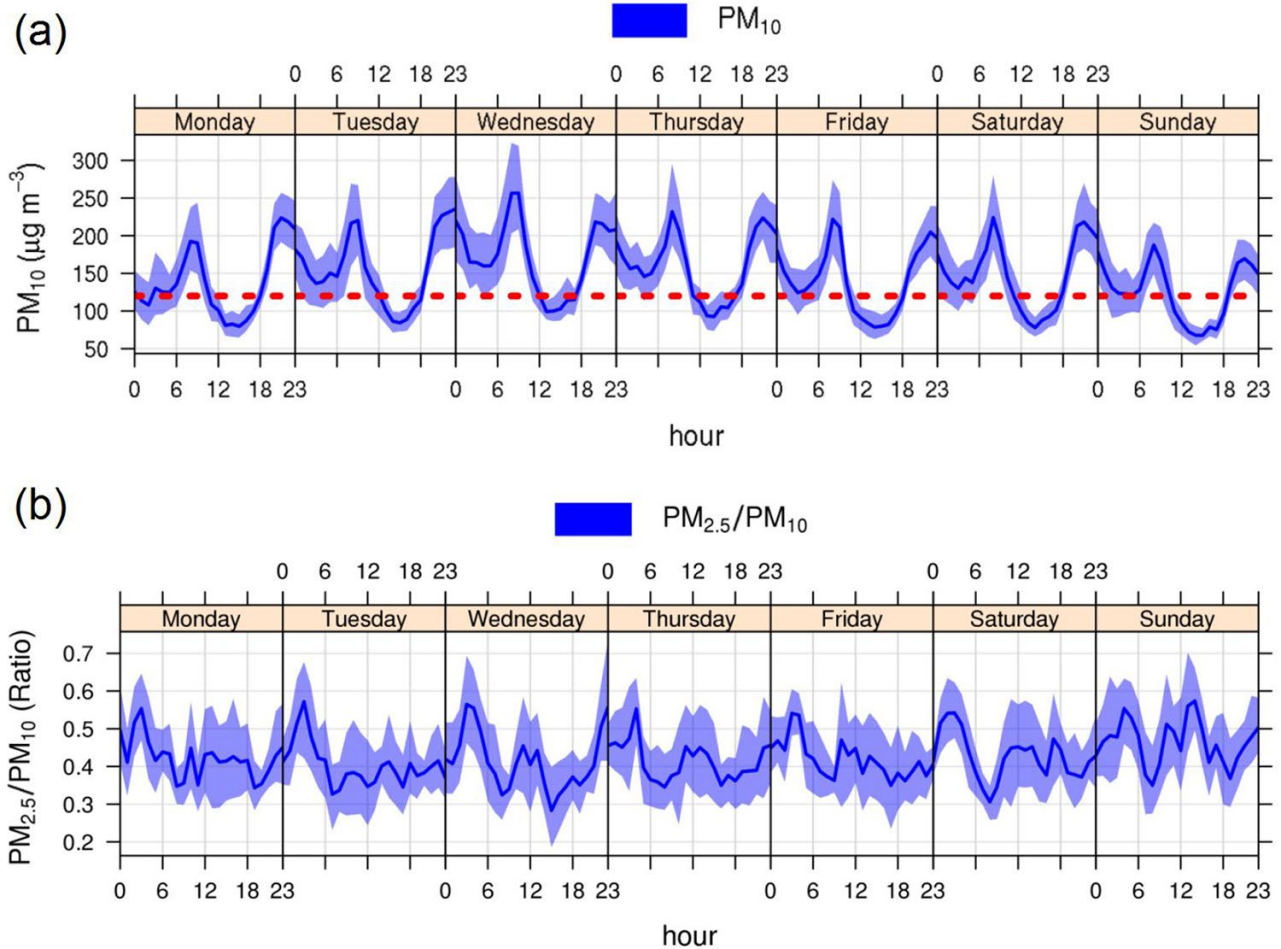
Variation of four year averages of (**a**) PM_10_ concentrations and (**b**) PM_2.5_/PM_10_ ratio on a daily basis in the selected winter months (November to February).

Figure 5 presents the diurnal variations of PM_10_ concentrations and PM_2.5_/PM_10_ ratios on weekends and weekdays in the selected winter months (November to February). Diurnal changes of PM_10_ for each month expressed the same trend of variations with substantially higher PM_10_ concentrations in January and February than that of the other months (Figure S3 in supplementary). It could be clearly seen that PM_10_ concentrations displayed two peaks daily. The first peak of PM_10_ began to rise at approximately 6:00 until 10:00, and decreased at noon. At around 19:00, PM_10_ concentrations started to rise again until about 23:00 resulting in the second peak observation.

The ratio of PM_2.5_/PM_10_ rising coincided with PM_10_ reduction such as at 3:00 whereas the ratio reducing coincided with PM_10_ increase such as at 9:00. The change of PM_10_ concentration varied mainly by the change of PM_coarse_. For other months, they showed the same characteristics, only the proportion of PM_coarse_ changed during the rainy season because large particles were removed from the atmosphere. Notably, the occurrence of the two peaks daily indicated the activities and factors related to the regular emission sources in the area for each day and month. In addition, these two peaks were consistent with Phetrawech and Thepanondh^14^ in that the emission rate of resuspended road dust in Na Phra Lan was high from 6:00–9:00 and 19:00–23:00.

### Potential sources of PM_10_

Identifying significant sources of PM_10_ is important for the policymakers to take appropriate action to mitigate and reduce these emissions. Bivariate polar plot technique can be used to identify a significant source^15,41,42^. The plot uses polar coordinates where the radial axis represents wind speed and the angular axis represents wind direction to display distributing concentrations of PM_10_ around the receptor. When wind speed is low, the mean concentrations will be displayed near the center of the polar coordinate, i.e., the receptor or the air quality monitoring station. However, when wind speed is high, the concentrations will be displayed far from the center. Polar plot results can be interpreted by comparing with the spatial map showing the locations of activities or sources related to PM_10_ emissions in the area.

**Figure 6:**
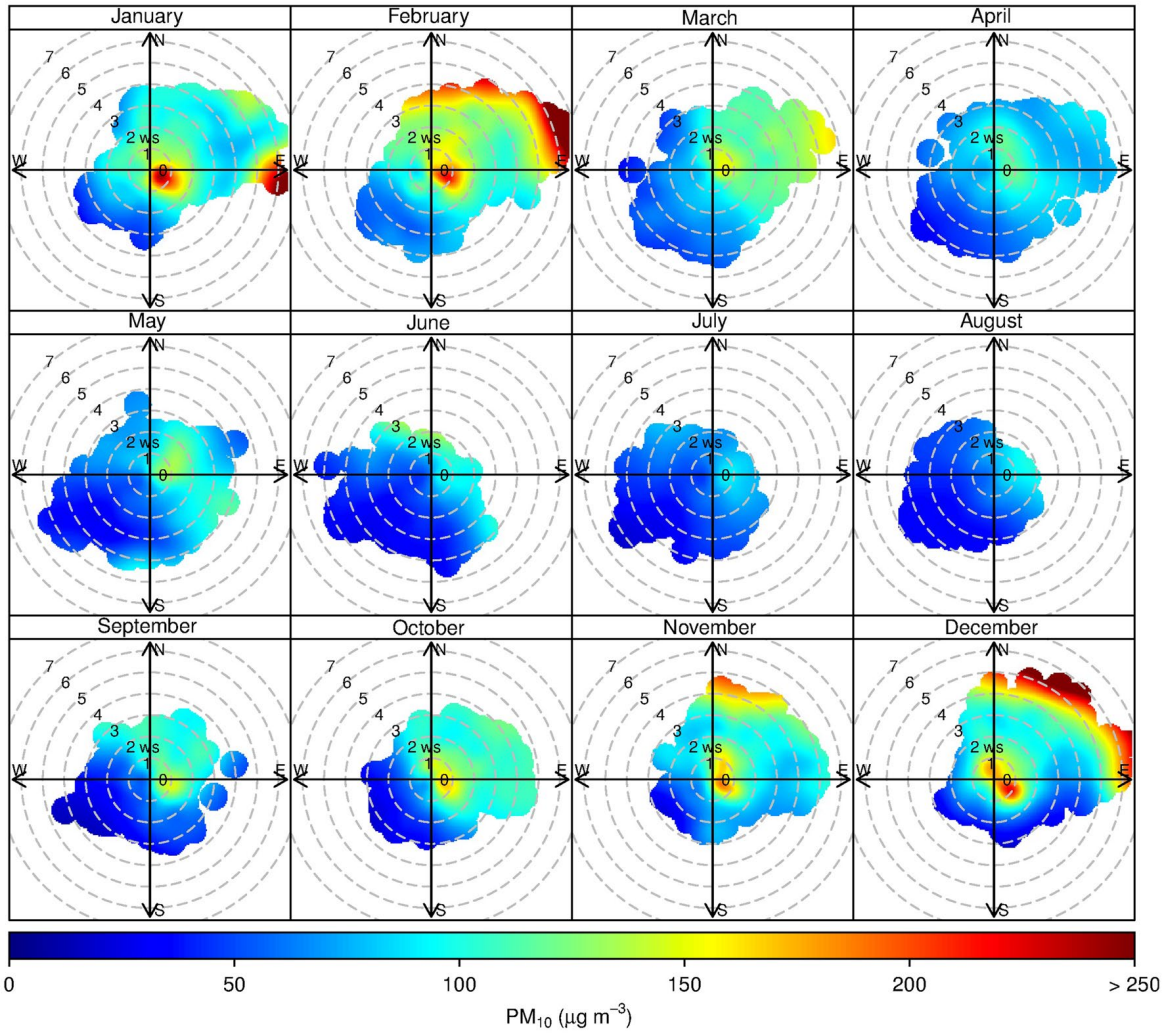
Bivariate polar plot of the mean PM_10_ concentration.

Figure 6 shows bivariate polar plot analysis based on 4 years of data on air quality and meteorological measurements at Na Phra Lan Subdistrict. High PM_10_ concentrations observed at the air quality monitoring station were identified at two main emission sources (red shading) in the north-east side from November to February. The first potential sources located far from the station corresponding to the low PM_2.5_/PM_10_ ratios (less than 0.5) (Figure S4 in supplementary). The low ratio was associated with more contribution of primary coarse particles^38^, mechanical grinding and crushing activities, and non-combustion sources, such as mining, quarrying, and agriculture^40^. As shown in Fig. 1, the far areas in the north-east side are mining and quarrying activities, which are around 1–5 km from the air quality monitoring station that agrees with this bivariate polar plot analysis results. Not only the far source has small PM_2.5_/PM_10_ ratio, but the close source is also. The second potential source is present near the air quality monitoring station, which is the roadside and this source is not representative of an urban traffic area. The small PM_2.5_/PM_10_ ratio would be emitted from soils by wind erosion^43^ and primary sources related to mechanical processes^38^. Therefore, the near source would be related to the resuspended road dust from transporting mining products as also suggested by Phetrawech and Thepanondh^14,44^ not tailpipe.

**Figure 7:**
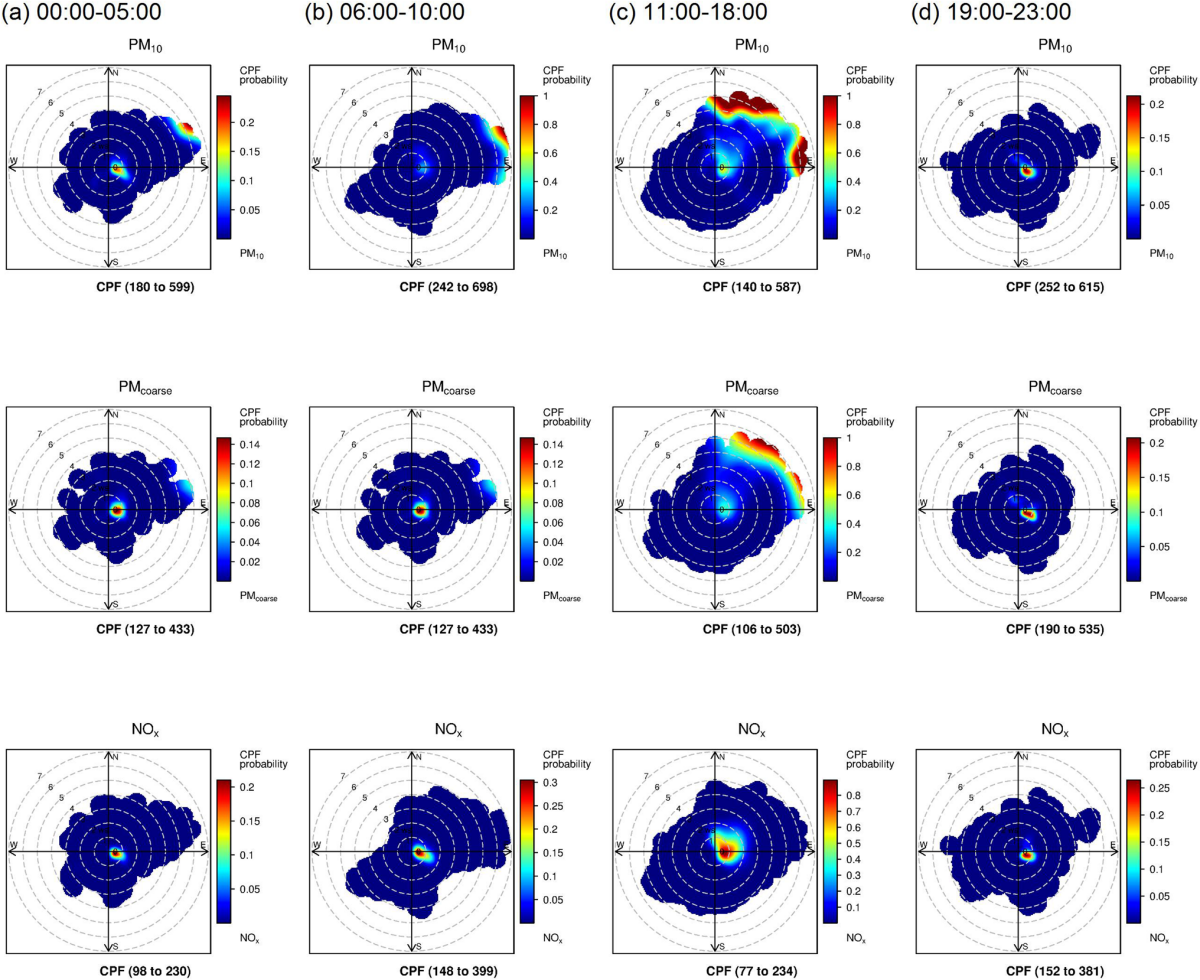
CBPF plot of PM_10_, PM_coarse_, NO_x_ concentrations for 91–100 percentile range at four periods (**a**) 00:00–05:00, (**b**) 06:00–10:00, (**c**) 11:00–18:00, (**d**) 19:00–23:00. (Range in the bracket is the concentration range in a unit of µg/m^3^ and ppb for PM and NO_x_, respectively).

The results obtained from the bivariate polar plot provide information in terms of spatial analysis for source dispersion; however, the polar plot cannot indicate the temporal influence of the source for each time window. Therefore, the CBPF plot was applied to analyse the four years data. The data were divided into four periods: 00:00–05:00, 06:00–10:00, 11:00–18:00, and 19:00–23:00, which were related to the two peaks and diurnal variation of PM_10_ concentration. During the period of diurnal low concentration from late night to morning, 00:00–05:00, the source area located near the air quality monitoring station and the far source in the Northeast direction influenced PM_10_ level (Fig. 7a). The potential source areas of PM_coarse_ were similar to the PM_10_ source areas; but, the potential source area of NO_x_ presented only the area located near the station. When the first peak of PM_10_ occurred in late morning (06:00–10:00), high PM_coarse_ concentration was found near the monitoring station. At the same time, the emission sources located far from the station started influencing PM_10_ levels (Fig. 7b). At noon (11:00–18:00), the sources located far from the station became the main contributor of PM_10_ at the station during the diurnal low concentration of PM_10_ (Fig. 7c). For the last period, evening to midnight, 19:00–23:00 (Fig. 7d), the second PM_10_ peak period, all PM_10_, PM_coarse_, NO_x_ emission source areas close to the station became the major contributor.

The results indicated that sources close to the air quality station substantially contributed to high concentrations of PM_10_ at the station from night to late morning and contributed to two peak levels of PM_10_ at 06:00–10:00 and 19:00–23:00. However, PM_10_ emission sources located far from the station were the main contributor from 11:00–18:00. The transportation source increased PM_10_ levels from 00:00 to 05:00 and triggered the first PM_10_ peak from 06:00–10:00. The increased PM_10_ was mostly contributed by PM_coarse_, which would be from the resuspended dust caused by transportation and the wind blowing from the mining and quarrying areas. The influence of mining and quarrying industries started contributing to PM_10_ level and became the main contributor of PM_10_ after 11:00 until 18:00. The increased level of PM_10_ at the air quality station during day time was affected by the dispersion from mining and quarrying areas to the station^13^. From 19:00–23:00, the second PM_10_ peak occurred, the main source being the transportation sector. The PM_10_ level in the second peak was mostly higher than the level of the first peak. The reason could be due to the stable condition of atmosphere and the reduced mixing layer height during the nocturnal time period resulting in limited vertical dispersion^13^. This mechanism of source emission and meteorology repeated on the next day and month.

This finding confirmed that the street located close to the station was an important source to trigger and increase the PM_10_ level by the resuspended dust more than emission of tailpipe from daybreak to late morning that resulted in PM_10_ peaks. The distant source from the industrial processes of mining and stone crushing in the north-east side maintained the background PM_10_ concentration in the area.

## Conclusion

The PM, NO_x_ and meteorological data observed at the air quality monitoring station located in Na Phra Lan Subdistrict, Saraburi Province, Thailand for the past four years were analysed to understand the air quality situation and characterise PM_10_ emission sources. From time series analysis, the mean concentration of PM_10_ in Na Phra Lan Subdistrict was higher than the NAAQS of Thailand and interim target-2 level of WHO mainly from October-February. The small PM_2.5_/PM_10_ ratio suggests that high PM_10_ concentration is caused more by PM_coarse_ than PM_2.5_, and related to primary sources, mining, and quarrying. The diurnal variation of hourly PM_10_ concentrations revealed two peak periods from 06:00–10:00 and 19:00–23:00 daily and monthly. This means PM_10_ levels exhibited similar daily behaviours contributed from the constant source emissions. Using bivariate polar plot and CBPF, the first potential source areas of PM_10_ were found at the far area in the northeast side. Most activities in this area were related to stone crushing, quarrying and mining industries. The second potential source was an area adjacent to the air quality monitoring station, in the area of the street. The small PM_2.5_/PM_10_ ratio presented over the near-source area implies less PM_2.5_; thus, the main source would be related to resuspended dust rather than the tailpipe emissions. Both of potential source areas played an important role in establishing the first PM_10_ peak. After that, the PM_10_ concentration decreased during the 11:00–18:00 and the far source area became the potential source. The near-source area, later on, contributed significantly in increasing PM_10_ by traffic acting to spread coarse dust in the later time during the nocturnal condition. The analysis with time series and CBPF could identify the mechanism and source that affected high PM_10_ concentration range in the specific location and temporal variations. From this study, we concluded that the industrial source of mining, stone crushing, and quarrying area played a role regarding background PM_10_ concentrations in Na Phra Lan Subdistrict and the mobile source was a factor to rebound PM_10_, particularly PM_coarse_, from the road to ambient air resulting in two PM_10_ peaks daily. To overcome the nonattainment area for PM_10_ attributable to the mining industrial and relating processes, we proposed that countermeasure activities, e.g., road cleaning before the peak times, change the route for transporting products from the mine and quarry, and improvement of industrial processes at the potential source area, particularly during October to February, would be required to reduce background PM_10_ levels.

## Supplementary Information

Supplementary Figures.
