# Supplementary material for: Characterising particulate matter source contributions in the pollution control zone of mining and related industries using bivariate statistical techniques
Source: Sci Rep. 2020 Dec 7;10:21372. doi: 10.1038/s41598-020-78445-5 (PMC7721878; doi:10.1038/s41598-020-78445-5)
Supplement: Supplementary file 1 — Supplementary Figures. [file 41598_2020_78445_MOESM1_ESM.pdf]

Supplementary

Characterising particulate matter source contributions in the pollution control zone of mining and related industries using bivariate statistical techniques

Sirapong Sooktawee [1], Thongchai Kanabkaew [2,3*], Suteera Boonyapitak [1], Aduldech Patpai [1], Nirun Piemyai [1]

[1] Environmental Research and Training Center, Department of Environmental Quality Promotion, Ministry of Natural Resources and Environment, Thailand
[2] Department of Sanitary Engineering, Faculty of Public Health, Mahidol University, Thailand
[3] Center of Excellence on Environmental Health and Toxicology, Thailand

*Correspondence to thongchai.kaa@mahidol.ac.th

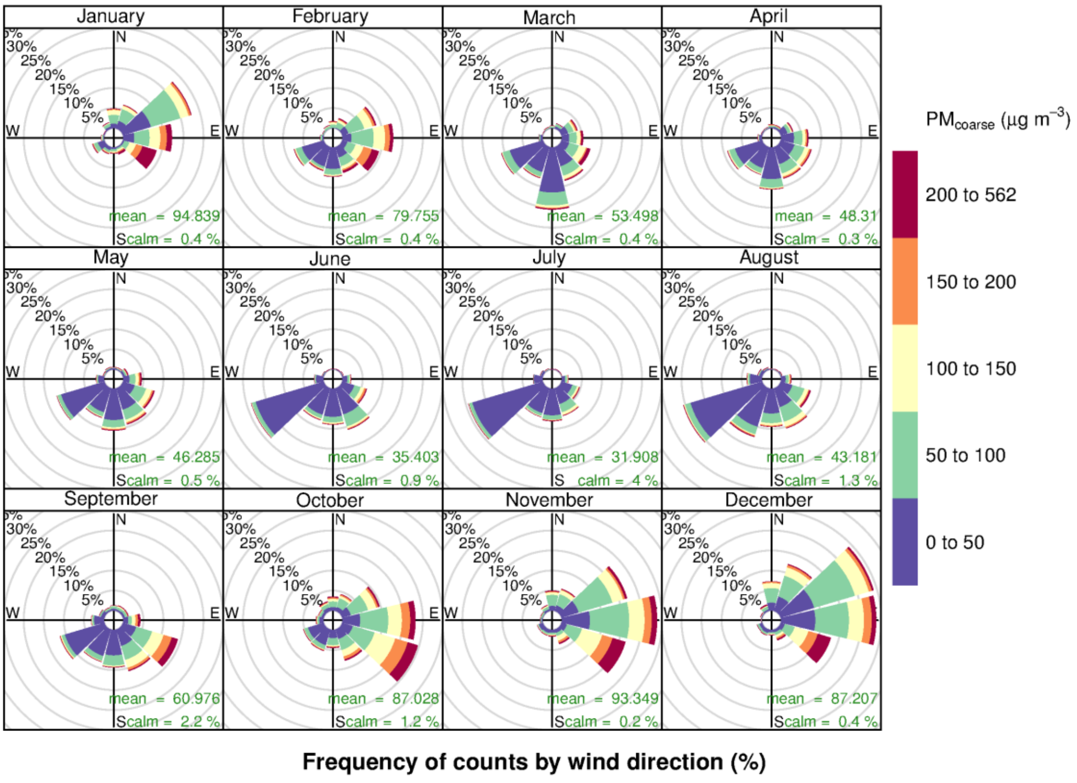

Figure S1. Monthly pollution roses of PM_{coarse} in Na Phra Lan Subdistrict.

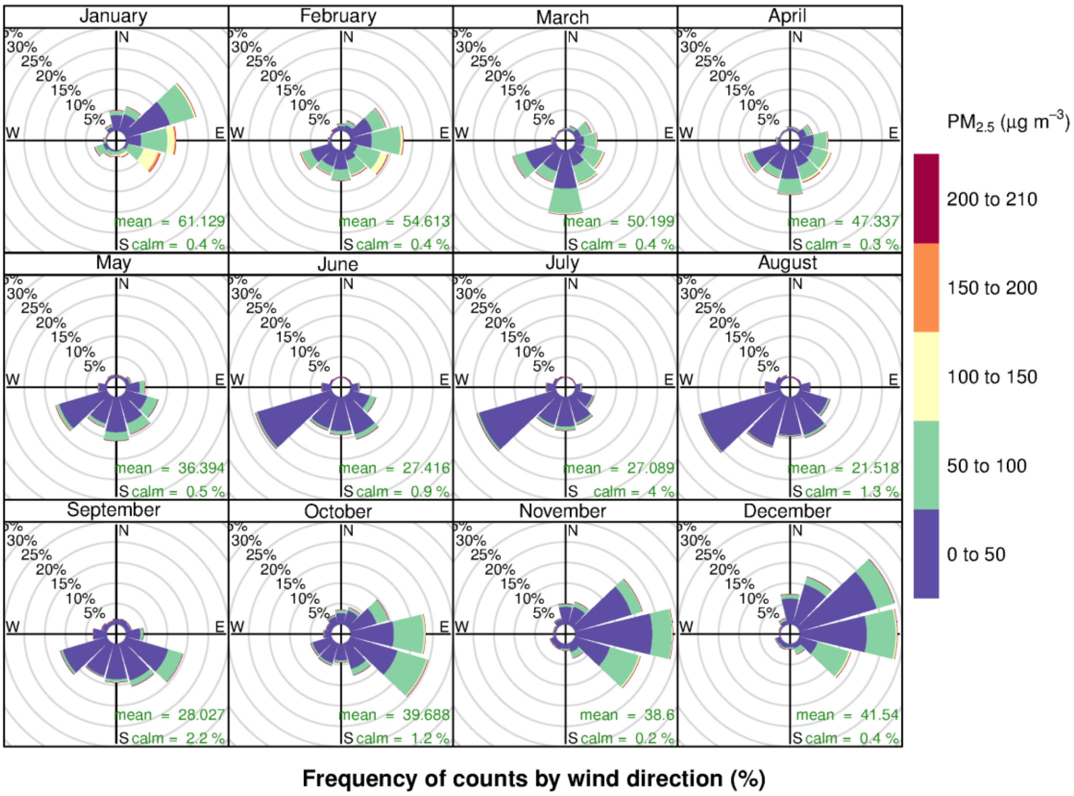

Figure S2. Monthly pollution roses of $PM_{2.5}$ in Na Phra Lan Subdistrict.

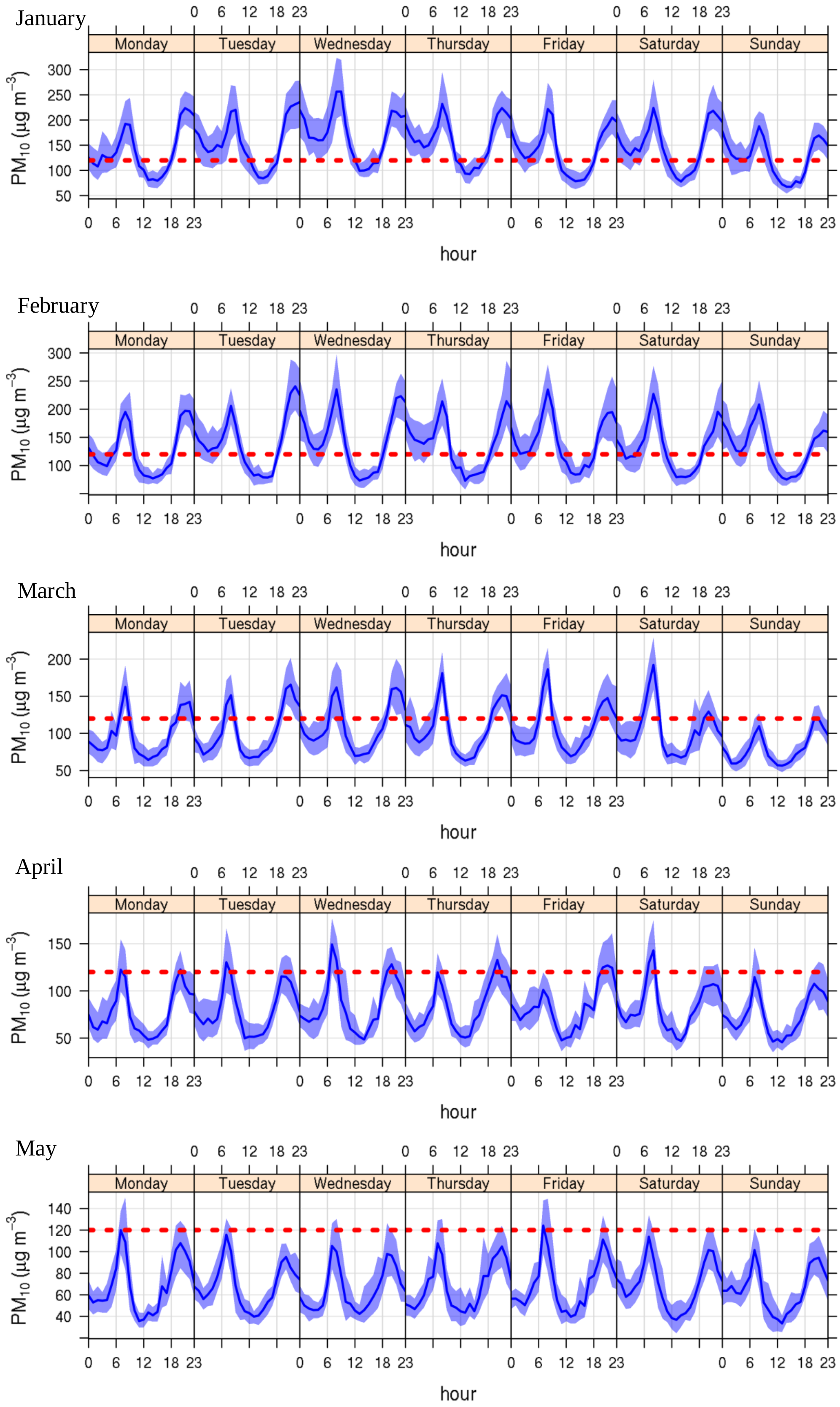

Figure S3. Variation of four year averages of PM_{10} concentrations on a daily basis for each month.

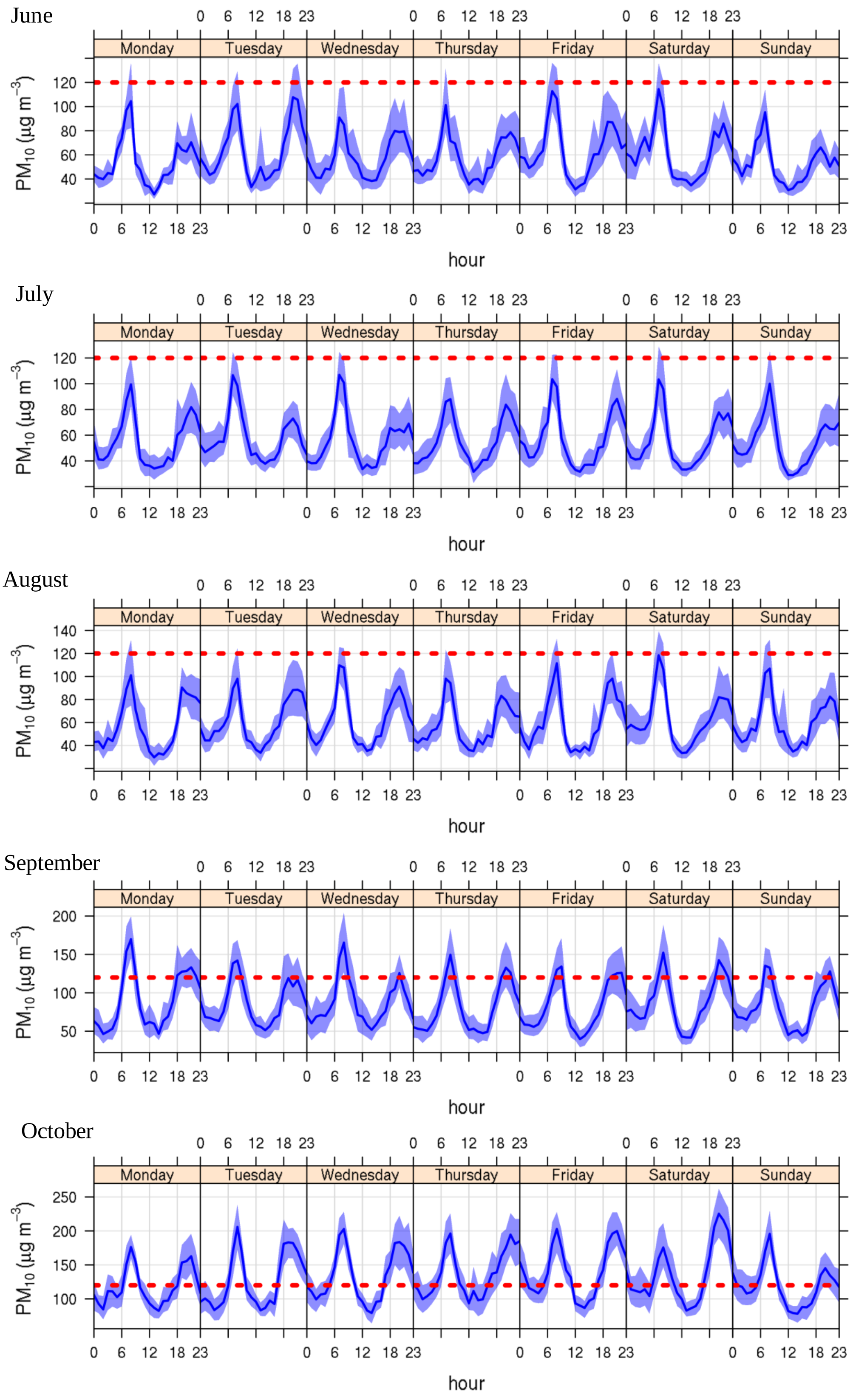

Figure S3. Continued

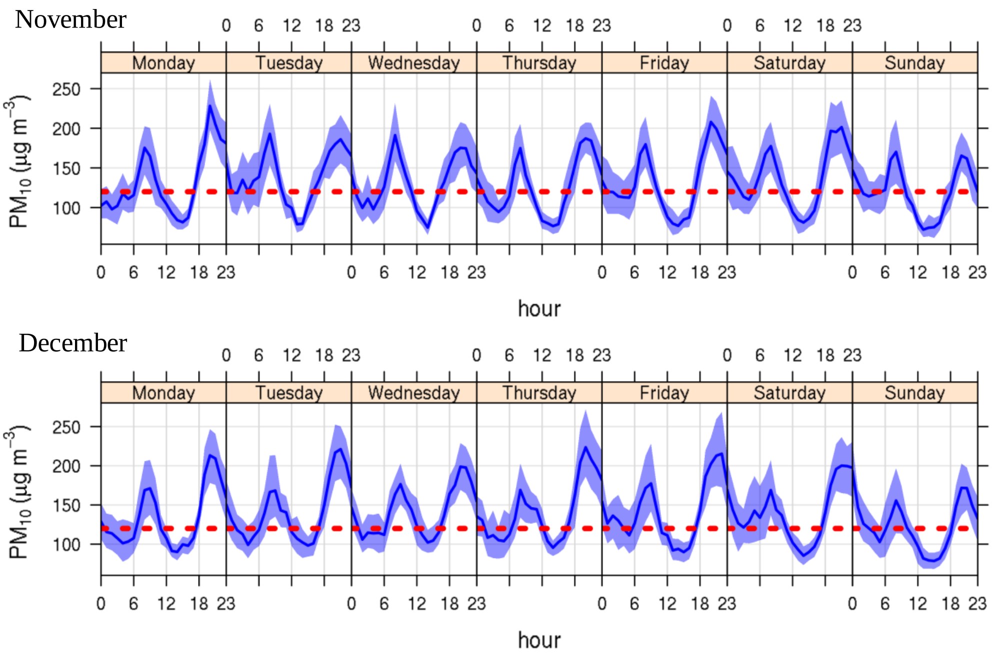

Figure S3. Continued

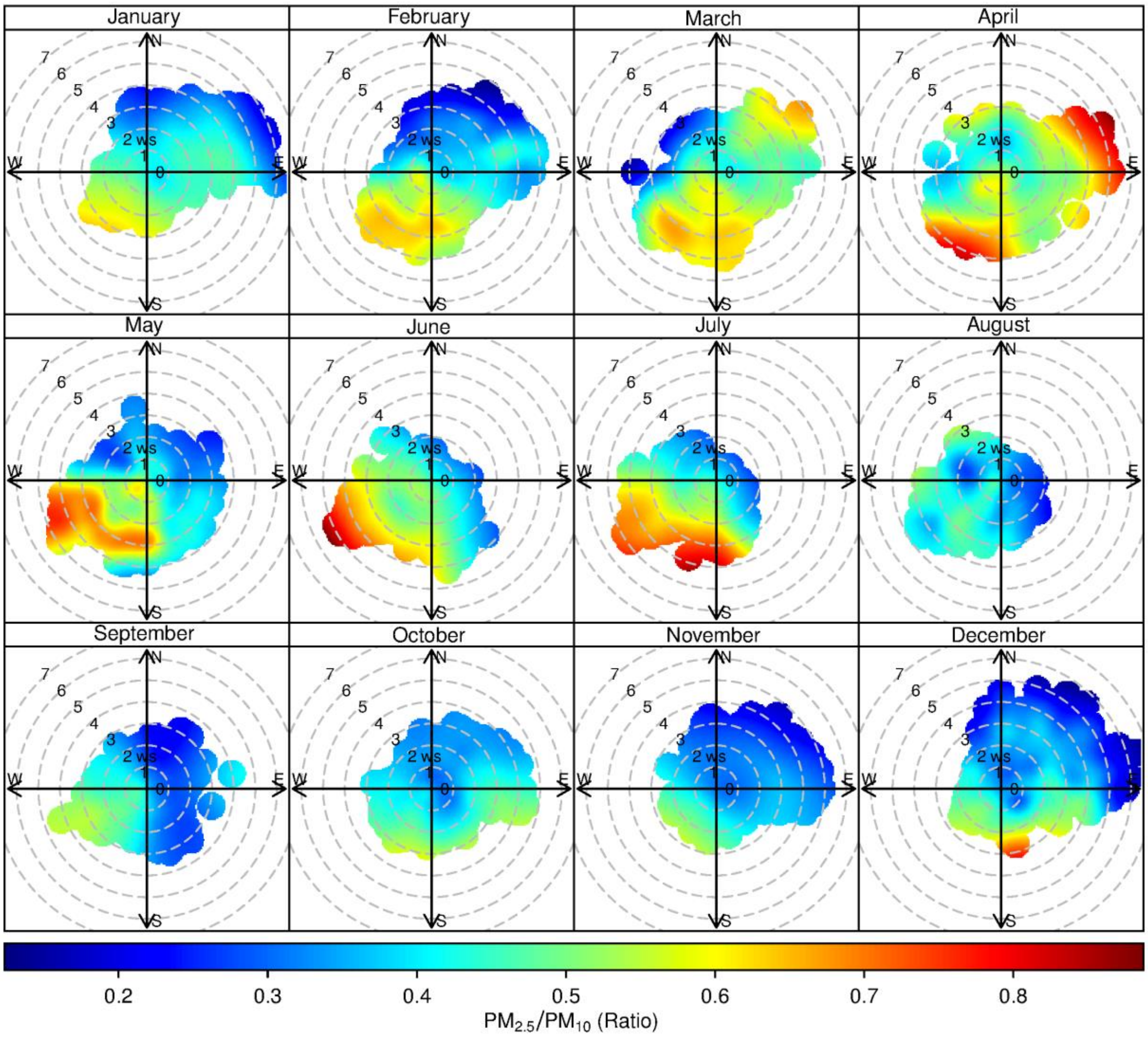

Figure S4. Bivariate polar plot of the mean $PM_{2.5}/PM_{10}$ ratio.
